# Observation of supersymmetric pseudo-Landau levels in strained microwave graphene

**DOI:** 10.1038/s41377-020-00351-2

**Published:** 2020-08-19

**Authors:** Matthieu Bellec, Charles Poli, Ulrich Kuhl, Fabrice Mortessagne, Henning Schomerus

**Affiliations:** 1 Institut de Physique de Nice (INPHYNI), Université Côte d’Azur, CNRS, 06108 Nice, France; 2 Department of Physics, Lancaster University, Lancaster, LA1 4YB UK

**Keywords:** Photonic crystals, Photonic devices, Optics and photonics

## Abstract

Using an array of coupled microwave resonators arranged in a deformed honeycomb lattice, we experimentally observe the formation of pseudo-Landau levels in the whole crossover from vanishing to large pseudomagnetic field strengths. This result is achieved by utilising an adaptable setup in a geometry that is compatible with the pseudo-Landau levels at all field strengths. The adopted approach enables us to observe the fully formed flat-band pseudo-Landau levels spectrally as sharp peaks in the photonic density of states and image the associated wavefunctions spatially, where we provide clear evidence for a characteristic nodal structure reflecting the previously elusive supersymmetry in the underlying low-energy theory. In particular, we resolve the full sublattice polarisation of the anomalous 0th pseudo-Landau level, which reveals a deep connection to zigzag edge states in the unstrained case.

## Photonics: Deepening our understanding of a material’s topological states

A deeper understanding of how the topological states of graphene-like structures affect their optical properties could pave the way for applications such as flat-band lasers and novel sensors. A material’s topological state can provide it with unusual properties. For example, topological insulators can act as insulators in their interior but allow currents to flow in opposite directions on their surface. Similar properties can also be engineered into optical systems. Combining theory led by Henning Schomerus from Lancaster University in the United Kingdom, and experiments carried out by Matthieu Bellec and Fabrice Mortessagne from Université Côte d’Azur in France, a team of researchers has demonstrated how the application of a deformation in a photonic analogue of graphene results in the formation of pseudo-Landau levels. The work shows that the material’s edge and bulk properties and sub-lattice polarisation are directly linked and provides a unifying principle that connects a wide variety of topological phenomena.

## Introduction

Topological states enjoy intense attention, as they equip quantum systems with desirable robust properties. Much of the early focus rested on their unique spectral positions as isolated or dispersive states in a band gap, as well as their spatial localisation at edges and interfaces^1^, or more recently also corners^2^. More fundamental characterisations, on the other hand, often invoke a third, somewhat deeper feature of topological states, which is connected to the anomalous expectation values of the underlying symmetry operators^3,4^. In momentum space, this feature underpins, e.g., the unidirectional chiral currents around the edges of topological insulators, while in real space, it manifests itself, e.g., in the sublattice polarisation of defect states in bipartite lattice systems^5^, as has been exploited in recent topological lasers or nonlinear limiters based on photonic Su-Schrieffer-Heeger structures^6–9^.

An additional attractive aspect of these anomalous features is that they tie topological effects together that are often seen as separate due to the varied nature of the specific encountered spectral and spatial features that first come into focus. A prime example are flat bands, which have been observed in recent experiments focusing on Lieb lattices^10–13^ and their one-dimensional counterparts^14^, as well as suitably deformed graphene^15,16^ and analogous quantum^17^ and classical systems^18,19^. In the latter case, these flat bands constitute pseudo-Landau levels arising from a synthetic magnetic field^20^. In particular, the signatures of photonic pseudo-Landau levels have been detected by probing the edges of a honeycomb array of optical waveguides^18^. A second example is a class of helical edge states in reciprocal systems, as observed, e.g., in zigzag terminated graphene^21,22^. While these bulk and edge phenomena do not naturally fall into the scope of standard topological band structure theory^1^, they are still intimately linked to wavefunctions with a characteristic sublattice polarisation. This association provides a promising perspective from which one can seek to develop very general unifying descriptions (see, e.g., Kunst et al.^23^ for a recent approach utilising this perspective).

In this work, we demonstrate experimentally for the case of photonic graphene-like systems that the anomalous edge and bulk phenomena tied to sublattice polarisation are in fact directly linked. This linkage is achieved by tracing the formation of the pseudo-Landau levels all the way from vanishing to large pseudomagnetic field strengths. In particular, we report the direct observation of the spatially resolved sublattice polarisation in the 0th pseudo-Landau level of strained photonic graphene and trace it back to the unstrained case, where the system only possesses edge states. By observing a characteristic nodal structure for the higher-order levels, we can then establish a direct link to the supersymmetric Hamiltonian of the underlying low-energy theory^24^. Therefore, our observations connect a broad variety of topological phenomena to a unifying principle.

## Results

### Microwave setup and optimal strain geometry

**Fig. 1:**
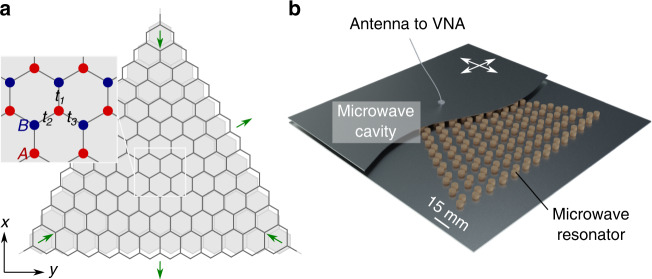
Experimental setup. **a** Sketch of the unstrained (grey) and strained (black) honeycomb lattice geometry, forming a zigzag terminated triangle of size *L* = 14. The green arrows indicate the directions ***ρ***_*l*_, *l* = 1, 2, 3, of the effective triaxial strain. Inset: the underlying lattice is composed of two sublattices A (red) and B (blue). The local coupling strengths are denoted *t*_*l*_ and depend on the location of the bonds in the lattice [see Eq. (1) and the text for details]. **b** Illustration of the experimental setup. The lattice is composed of 196 identically designed cylindrical dielectric resonators that are coupled through the evanescent field of the fundamental TE mode. The structure is placed inside a microwave cavity made of two metallic plates (top plate only partially shown). A loop antenna mounted on a scanning system (white arrows) crossing the top plate and connected to a vector network analyzer (VNA) is used to generate and collect the spectrally and spatially resolved microwave signal, which allows us to obtain the local density of states in the system

Our experimental setup is illustrated in Fig. 1. The unstrained system forms a honeycomb lattice with nearest-neighbour spacing *a*_0_ = 13.9 mm, combining two triangular lattices of A and B sites, where each vertex denotes the position of a dielectric microwave resonator with bare frequency *ω*_0_ = 6.653 GHz, while adjacent resonators are coupled at strength *t*_0_ = 21.5 MHz (for details see the materials and methods section). This configuration gives rise to a standard graphene-like photonic band structure^25,26^, with two Dirac cones at the *K* and *K*’ points in the Brillouin zone. Around these two so-called valleys, at relative momentum ***q***, the low-energy dispersion $\omega \left( {\it{q}} \right)\sim \omega _0 \pm v\left| q \right|$ resembles massless relativistic particles moving in two dimensions at velocity *v* = 3*a*_0_*t*_0_/2.

In the deformed system, the indicated couplings *t*_*l*_ depend on the distances to the three neighbouring resonators, which can be utilised to create a pseudomagnetic field corresponding to that in strained graphene. Such a field arises when the resonators are displaced nonuniformly, where the positions are selected to give a triaxial spatial coupling profile^20,27^

1 $$t_l = t_0\left[ {1 - \frac{\beta }{{2a_0^2}}{\it{\uprho }}_l \cdot {\it{r}}} \right]$$

where ***r*** refers to the positions of the links between the coupled resonators in the unstrained system and the bond vectors ***ρ***_*l*_ are pointing along these coupling directions. This coupling profile ensures a constant pseudomagnetic field of strength *β* throughout the whole system. Theoretically, the system is well described in a coupled-mode theory with nearest-neighbour couplings *t*_*l*_ as given above, so that the eigenfrequencies and mode profiles can be obtained from an effective Hamiltonian *H*. As with any such bipartite system, it then displays a chiral symmetry relative to the central frequency, ∑_*z*_(*H*−*ω*_0_)∑_*z*_ = −(*H*−*ω*_0_), where the Pauli-like matrix ∑_*z*_ acts on the sublattice degree of freedom, hence keeping the amplitudes on the A sites fixed but inverting those on the B sites. The balance of zero modes on the A and B sublattices is given by the signature of this operator^13,28^,

2 $$\# \left( {{\mathrm{A}}\,{\mathrm{zero}}\,{\mathrm{modes}}} \right)-\# \left( {{\mathrm{B}}\,{\mathrm{zero}}\,{\mathrm{modes}}} \right) = {\mathrm{tr}}\,{\Sigma} {\,_z} = \# \left( {{\mathrm{A}}\,{\mathrm{sites}}} \right)-\# \left( {{\mathrm{B}}\,{\mathrm{sites}}} \right)$$

These zero modes have frequency *ω*_0_ and, as indicated, are localised on a given sublattice. Furthermore, the chiral symmetry dictates that all nonzero modes occur in spectral pairs at symmetric positions *ω*_0_ ± *δω* and have equal intensity on both sublattices.

Importantly, in contrast to earlier experimental work, we select a triangular geometry and terminate the system with zigzag edges. This configuration ensures a number of beneficial features^29,30^. Particularly relevant for us, the boundary conditions are then compatible with the bulk pseudo-Landau levels at all field strengths; furthermore, a consistent coupling profile can be maintained even at maximal field strength, whose description requires going beyond the conventional low-energy analogy to magnetic fields with opposite signs in the two valleys. Setting the pseudomagnetic field strength to a fixed value, we then see that the values of the couplings dictated by profile (1) drop to zero exactly at the terminating edges of a zigzag terminated triangle. The corresponding maximal field strength is *β*_*M*_ = 4/*L*, where the size parameter *L* counts the terminating A sites along each edge.

In the experiments, we realise these conditions in a system with 196 resonators, corresponding to a triangle with *L* = 14 resonators along each terminating edge (see Fig. 1). Of these, 105 resonators are on the A sublattice, while 91 are on the B sublattice. These conditions allow us to realise a field strength up to *β* = 0.2, where the extremal couplings still exceed the homogeneous resonator linewidth *γ* = 1.7 MHz, and is sufficiently close to *β*_*M*_ to clearly demonstrate the detailed features of well-formed pseudo-Landau levels. To obtain the analogous orbital effects for an electron in graphene, a magnetic field of 42000 *T* would have to be applied.

### Formation of pseudo-Landau levels

**Fig. 2:**
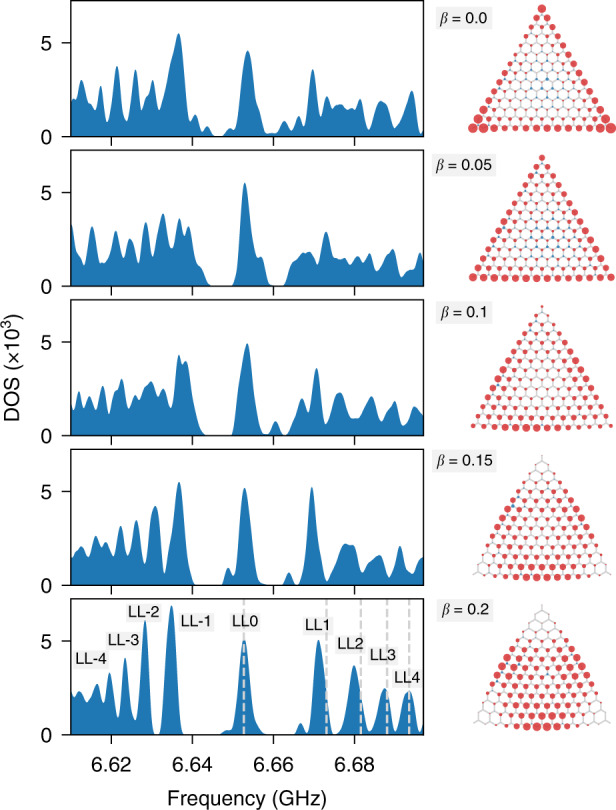
Landau-level formation. Experimentally determined density of states (left panels) and spatially resolved mode intensity associated with the central peak centred at *ω*_0_ = 6.653 GHz (right panels, obtained by integrating the local density of states over the peak). The area of the circles corresponds to the intensity on the A sites (red) and B sites (blue). From top to bottom, the pseudomagnetic field strength *β* varies from 0 to 0.2. The grey dashed lines in the total density of states for *β* = 0.2 depict the expected pseudo-Landau level frequencies from coupled-mode theory. The formation of the 0th pseudo-Landau level proceeds continuously by transforming the zigzag edge states into bulk states while retaining the degeneracy (spectral weight) and sublattice polarisation (remaining confined on the A sublattice)

Figure 2 shows our main experimental results. The panels on the left show the density of states for the resonator geometry on the right, where each row corresponds to a different value of the pseudomagnetic field strength *β*. The colour density plot overlaid with the resonator lattice depicts the local density of states integrated over the central peak, situated at the bare resonator frequency *ω*_0_.

In the pristine system (*β* = 0), this peak arises from the zigzag edge states, which are localised on the terminating resonators. Note that these resonators all occupy the same sublattice of the A sites. On this sublattice, the zigzag states decay into the bulk of the system, while they maintain a vanishing density on the B sublattice. The simple rule (2) can be exploited to count the number of these zigzag states: as a consequence of the chiral symmetry of the system, this number is expected as the difference 105−91 = 14 = *L* of A and B sites in the system. Away from the central peak, the density of states displays a broad continuum, with fluctuations arising from the finite-size quantisation of bulk graphene-like states, including the states near the Dirac cones.

As the pseudomagnetic field strength is increased, we observe that the spectral weight from this continuum gradually reorganises into a sequence of well-defined peaks, which at large field strength obtain a similar width and weight as the central peak. Furthermore, while the weight and position of the central peak itself remain essentially unchanged, the spatial profile of the associated zero-modes changes significantly in that they move into the bulk, where they form the desired 0th pseudo-Landau level.

The observed spectral positions of the emerging higher-order pseudo-Landau levels conform well with the characteristic square-root dependence of relativistic Landau levels^24,31^, revisited later in the text and depicted by the grey dashed lines in Fig. 2. The same applies to the observed spectral weights. As the transformation from the zigzag edge states to the 0th pseudo-Landau level is continuous, this bulk level retains the same spectral degeneracy and hence here consists of *L* = 14 modes. The coupled-mode theory predicts that the *n*th pseudo-Landau level encompasses $14 - \left| n \right|$ states, which explains the gradual drop of the observed spectral weights of the peaks moving outwards from the central peak. Note that this feature implies an important difference from Landau levels arising from a magnetic field, for which the degeneracy is dictated by the sample area ∝ *L*^2^, but not the linear size *L*, as observed here and underpinned by general theory^29,30^.

### Supersymmetric nodal profiles

**Fig. 3:**
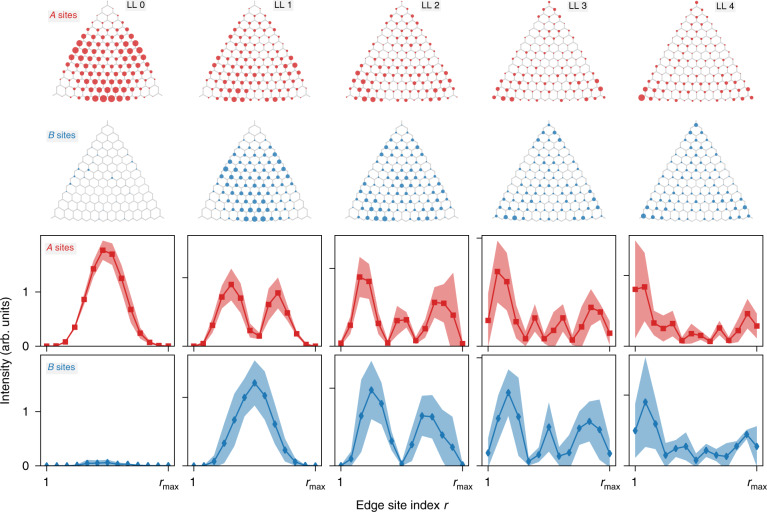
Supersymmetric node structure. Experimentally determined spatially resolved mode intensities associated with the pseudo-Landau levels of index *n* = 0–4 (from left to right) for a large pseudomagnetic field strength *β* = 0.2. Top rows: the area of the circles corresponds to the intensity on the A sites (above) and B sites (below). The 0th level is almost entirely localised on the A sublattice, while all other levels have almost equal overall weights on both sublattices. Bottom rows: intensity at sites A (above) and B (below), averaged along the three extreme edges at resonator position *r*. Note that *r*_max_ = 14 (resp. 13) for the A (resp. B) sites. For the higher-order levels, the average is performed combining the indices ±*n*. The shaded area corresponds to the standard deviation. The two *y*-axis ticks indicate arbitrary intensity values of 0 and 1 in uniformly applied units. The pseudo-Landau levels display a clear nodal structure offset by 1 mode index, as further discussed in the text

As anticipated, a notable feature in the formation of the 0th pseudo-Landau level verified in the experiment is the observation that across the whole transition, the associated modes remain localised on the A sublattice. In contrast, the modes in the emerging higher-order levels, while also localised in the bulk, are anticipated to display an equal weight on both sublattices. We analyse this distinction in detail in Fig. 3. The top rows display the experimental local density of states in the spectral range of the pseudo-Landau levels for the indices *n* = 0, 1, 2, 3, 4, all taken at the large pseudomagnetic field strength *β* = 0.2. As above, the level with *n* = 0, shown in the left panels, is located on the A sublattice. The higher-order levels shown in the other panels indeed display an approximately equal weight on both sublattices. Furthermore, these levels have an intensity profile that increasingly seeps into the corner areas of the triangle, which remains in line with the predictions of coupled-mode theory where the states form a complete basis.

The other two rows in Fig. 3 show the local density of states along the edges of the system separately for the A and B sublattices, where we averaged over the three edges and indicate the range of observed values by the shaded areas. For the higher-order levels, we further include the levels with index −*n* into the average. Along each edge, we observe standing-wave patterns with a characteristic nodal pattern represented by oscillating functions

3 $$\psi _r^{\left( {{\mathrm{A,}}\,{\mathrm{edge}}} \right)} \propto {\mathrm{sin}}\left[ {\left( {\left| n \right| + 1} \right)\pi r/\left( {L + 1} \right)} \right]$$

for resonator *r* = 1, 2, 3,…, *L* on the A sublattice, and a corresponding pattern

4 $$\psi _r^{\left( {{\mathrm{B,}}\,{\mathrm{edge}}} \right)} \propto {\mathrm{sin}}\left( {\left| n \right|\pi r/L} \right)$$

for resonator index *r* = 1, 2, 3,…, *L*−1 on the B sublattice.

The key observation is that the mode index $\left( {\left| n \right| + 1} \right)$ vs. $\left| n \right|$ in these patterns for the two sublattices is offset by one, which directly translates into the same offset of the number of nodal points along each edge. As we show in the supplemental material via the numerical modelling of the system in a coupled-mode tight-binding approximation, these nodal patterns persist for larger systems, with the only difference being an emerging modulation of the peak heights and spacings across the edge. An explicit construction of the edge states at maximal strain, also given in the supplemental material, reveals that these edge states indeed approach the bound-state sequence of a harmonic oscillator, with the corresponding sequences on the A and B sublattices offset by one pseudo-Landau level.

We now explain how these offset nodal patterns reveal the underlying supersymmetry of the pseudo-Landau levels. This supersymmetry is encoded in the low-energy theory of the system, which is formulated as a continuum approximation for energies close to the central frequency *ω*_0_. Relative to this central frequency, the pseudo-Landau levels are then described by an effective Hamiltonian^24,27,31^

5 $$H_0 = \left( {\begin{array}{*{20}{c}} 0 & {\pi ^\dagger } \\ \pi & 0 \end{array}} \right)$$

where the Landau-level annihilation and creation operators *π* and $\pi ^\dagger$ fulfil $\left[ {\pi ,\pi ^\dagger } \right] = 2v^2\beta /a_0^2$ (we assume *β* > 0; for opposite deformation, the operators *π* and $\pi ^\dagger$ interchange their roles and become creation and annihilation operators, respectively). This expression corresponds to the Hamiltonian of a relativistic electron in a magnetic field, which is formally identical to a supersymmetric harmonic oscillator^24,32^. The link to the offset nodal patterns along the edges arises from the fact that $H_0^2 = {\mathrm{diag}}\left( {\pi ^\dagger \pi ,\pi \pi ^\dagger } \right)$ factorises into two equidistant level sequences $E_n^2 = 2v^2n\beta /a_0^2$ on the two sublattices, where *n* = 0, 1, 2, 3,… on the A sublattice, while on the B sublattice *n* = 1, 2, 3,…, so that the sequence is indeed offset by one. At the edge of the system, the creation operator $\pi ^\dagger$ that connects these level sequences increases the number of nodes with each application by exactly one, so that the number of nodes coincides with the position of the level within the given symmetry sector of the theory. As mentioned above, this picture is further supported beyond the low-energy theory by the explicit construction of edge states given in the supplemental material. The experimentally observed offsets in the level sequence and the nodal patterns therefore both arise from a common origin.

## Discussion

We achieved the direct observation of the formation of pseudo-Landau levels in deformed honeycomb systems, both spectrally and in terms of their key spatial features. In particular, adopting a flexible dielectric-resonator array design with a purposefully selected geometry allowed us to follow the formation of these levels in the transition from vanishing to large pseudomagnetic field strengths. In this way, we could observe how the 0th pseudo-Landau level originates from the transformation of zigzag edge states into bulk states while maintaining its characteristic anomalous polarisation on only one of the two sublattices in the system. Extending these considerations to the higher-order levels allowed us to reveal a characteristic nodal structure of the pseudo-Landau level sequence that reflects the supersymmetric structure of the underlying low-energy description. These features underline the general usefulness of accounting for anomalous expectation values to provide a more general perspective on topological states.

Resolving the reported features in an experiment poses a significant challenge. In electronic systems such as graphene, spectral imaging techniques do not provide the required atomistic resolution, so that the information can only be extracted indirectly, for example, from the Fourier transformation of form factors^33,34^. A better resolution is offered by photonic systems^18^, which, however, so far, could not access the characteristic spatial features of the pseudo-Landau levels in the bulk of the system and furthermore considered a geometry that does not enable the observation of fully formed flat-band pseudo-Landau levels^29^. Drawing on an acoustic analogue^35^, a recent experiment^19^ managed to excite a compacton-like state in the 0th Landau level, demonstrating its characteristic sublattice polarisation in the bulk^27^. Here, we exploited an adaptable dielectric microwave-resonator array geometry to provide a complete characterisation of the system from the unstrained to the fully strained case. This characterisation allows us to reveal how zigzag edge states transform into the bulk states of the anomalous 0th pseudo-Landau level, where they retain their characteristic sublattice polarisation. Furthermore, this perspective dictates a natural geometry in which maximal pseudomagnetic fields can be attained and for which the pseudo-Landau levels remain compatible with the boundary conditions at all field strengths, in contrast to the previous experiments. This approach is required to obtain pseudo-Landau levels that are flat, which we demonstrate spectrally by the observation of sharp peaks in the photonic density of states, and enables us to reveal the supersymmetric signatures in the nodal structure.

Our results extend to a wide variety of flat-band systems, such as the rich physics arising from higher-order resonator modes in deformed honeycomb lattices, as reported for exciton polaritons in ref. ^36^. Practically, the observed spatial features should help to pave the way to applications such as flat-band lasers^13,27,37^, as well as sublattice-dependent sensors where one could exploit that chiral-symmetry breaking perturbations equip the 0th Landau level with a finite weight on the opposite sublattice.

## Materials and methods

### Experiment

The experimental setup is designed to realise a microwave system that is well approximated by a nearest-neighbour tight-binding description^26^. The sites of the lattice are occupied by dielectric microwave resonators with a cylindrical shape and are made of ZrSnTiO ceramics (Exxelia Temex, Paris, France, E2000 series: 5 mm height, 8 mm diameter and a refractive index *n* ≈ 6) sandwiched between two metallic plates at a distance *h* = 16 mm. Each resonator supports a fundamental transverse electric (TE) mode of bare frequency *ω*_0_ = 6.653 GHz, which corresponds to the on-site energy of atoms in a tight-binding model. Due to ohmic losses in the dielectric material and the metal, the quality factor of this mode is $Q \simeq 6000$, leading to a resonance width $\Gamma \simeq 5\,{\mathrm{MHz}}$. As the resonance frequency is below the cut-off frequency of the first TE mode defined by the two plates, the adjacent resonators are coupled through evanescent wave components, leading to an approximately exponential decay of the coupling strength *t* with the distance between the resonators^26^. The system is excited via a loop antenna fixed in the movable top plate, thus allowing the spatial scanning of the magnetic field *B*_z_, which is the only magnetic field component for this mode^38^. From the reflection measurements performed by a vector network analyzer (ZVA 24 from Rohde & Schwarz), the local density of states can be extracted (for details see ref. ^26^), and finally, by integrating over space, the density of states. In all these experiments, we face an intrinsic on-site disorder of ~0.15% in the values of *ω*_0_.

### Modelling

For the design of the system, we modelled finite strained photonic honeycomb lattices with a range of system sizes and boundary geometries within coupled-mode tight-binding theory, in which we accounted for the fundamental TE mode and incorporated the experimental distance dependence of the coupling strengths^26^. Exact diagonalization gives us access to the resonant modes and their spatial intensity distribution. The modelling confirmed that the triaxial coupling profile (1) can be attained by the suitable positioning of the resonators, resulting in an excellent match with the continuum theory predictions for the lowest Landau levels in systems as used in the experiments. The modelling further confirmed that only for zigzag boundaries can one attain the full sublattice polarisation of the 0th Landau level, and only for a triangular shape can the Landau levels become maximally degenerate.

## Supplementary Information

Supplementary Information
