# Supplementary material for: Observation of supersymmetric pseudo-Landau levels in strained microwave graphene
Source: Light Sci Appl. 2020 Aug 19;9:146. doi: 10.1038/s41377-020-00351-2 (PMC7438506; doi:10.1038/s41377-020-00351-2)
Supplement: Supplementary file 1 — Supplementary Information [file 41377_2020_351_MOESM1_ESM.pdf]

Observation of supersymmetric pseudo-Landau levels in strained microwave graphene

Supplemental Material

Matthieu Bellec*,[1] Charles Poli,[2] Ulrich Kuhl,[1]
Fabrice Mortessagne*,[1] and Henning Schomerus*[2]
[1]*Université Côte d'Azur, CNRS, Institut de Physique de Nice (INPHYNI), 06108 Nice, France*
[2]*Department of Physics, Lancaster University, Lancaster, LA1 4YB, United Kingdom*
*matthieu.bellec@inphyni.cnrs.fr, Fabrice.Mortessagne@unice.fr,
h.schomerus@lancaster.ac.uk

We here provide additional results for the edge nodal patterns based on tight-binding simulations of the strained honeycomb system, as well as an explicit analytical derivation of these patterns at maximal strain.

1. Numerical results

The numerical simulations are performed using a homemade Python package (tbee), developed to build and solve very general tight-binding models with potentially also complex-valued onsite energies and hoppings[1].

a. Formation of pseudo-Landau levels

Fig. S1 shows the spectrum and the corresponding zero-energy wavefunction intensities for various pseudomagnetic strains β ranging from 0 to the maximal value $\beta_M = 4/N$, for a system with $N = 28$ sites along a single edge. The emergence of the pseudo-Landau levels as β increases, accompanied with the transition from edge to bulk zero-energy states, is in very good agreement with the experiments (see Fig. 2 in the main text).

b. Nodal structure of the pseudo-Landau levels

Fig. S2 to S6 focus on the nodal structure of the pseudo-Landau levels at the maximal strain $\beta = \beta_M$. The spatial intensity distribution on each sublattice A and B of the nth pseudo-Landau level, n ranging from 0 to 4, is presented in Fig. S2 for a system with $N = 28$. The

corresponding intensities along the bottom edge are plotted in Fig. S4. The expected nodal pattern is observed: the mode index $(n+1)$ vs n in these patterns for the two sublattices is offset by one, which directly translates into the same offset of the number of nodal points along each edge. As shown in Fig. S3 to S6, these nodal patterns persist for different system sizes, (N from 14 to 70 respectively), with the only difference being an emerging modulation of the peak heights across the edge as the system size increases.

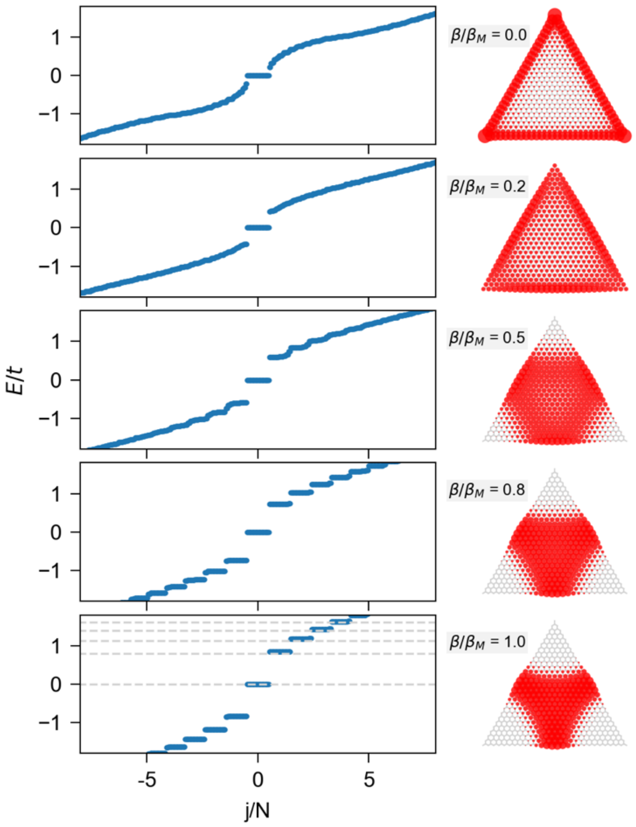

Figure S1. Landau-level formation. Eigenvalues (left panels) and zero-energy wavefunction intensities (right panels) for system size (number of bottom edge sites) $N = 28$. The area of the red circles corresponds to the intensity on the A sites (red), whilst the intensity on the B sites exactly vanishes. From top to bottom, the pseudomagnetic field strength β varies from 0 to $\beta_M = 4/N$. The gray dashed lines in the spectrum for $\beta = \beta_M$ indicate the expected position pseudo-Landau energy levels at maximal strain.

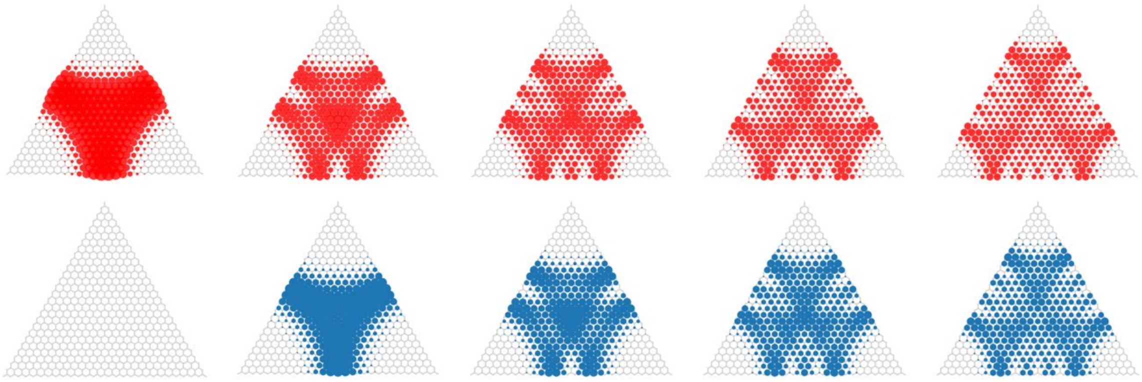

Figure S2. Supersymmetric node structure. Numerically obtained wavefunction intensities associated with the pseudo-Landau levels of index $n = 0$ to 4 (from left to right) for the maximal pseudomagnetic field strength $\beta = \beta_M$. In the top row, the area of the circles corresponds to the intensity on the A sites, whilst in the bottom row they correspond to the intensity on the B sites. The system size is $N = 28$.

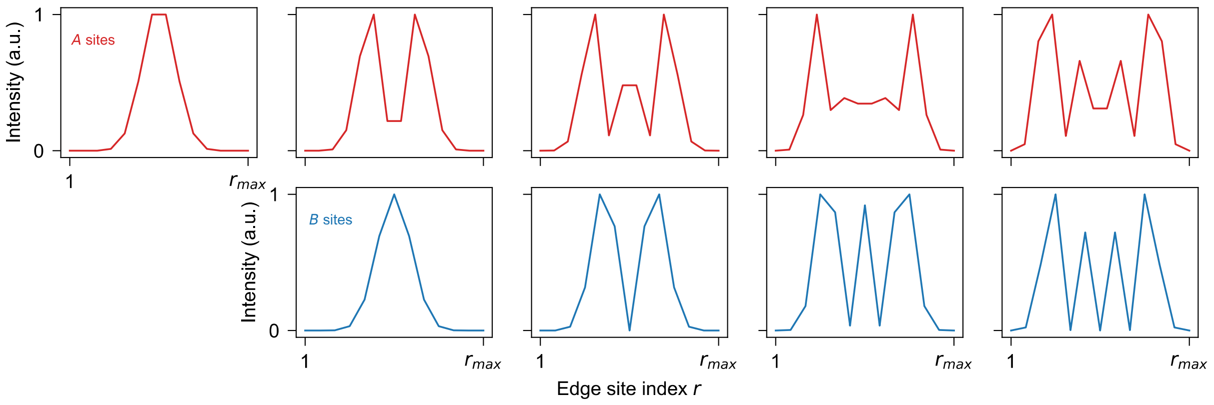

Figure S3. Intensity on A sites (top) and B sites (bottom) along the extreme bottom edge at resonator position r, for a system of size $N = 14$. Note that $r_{\max} = N$ for A sites and $r_{\max} = N - 1$ for the B sites.

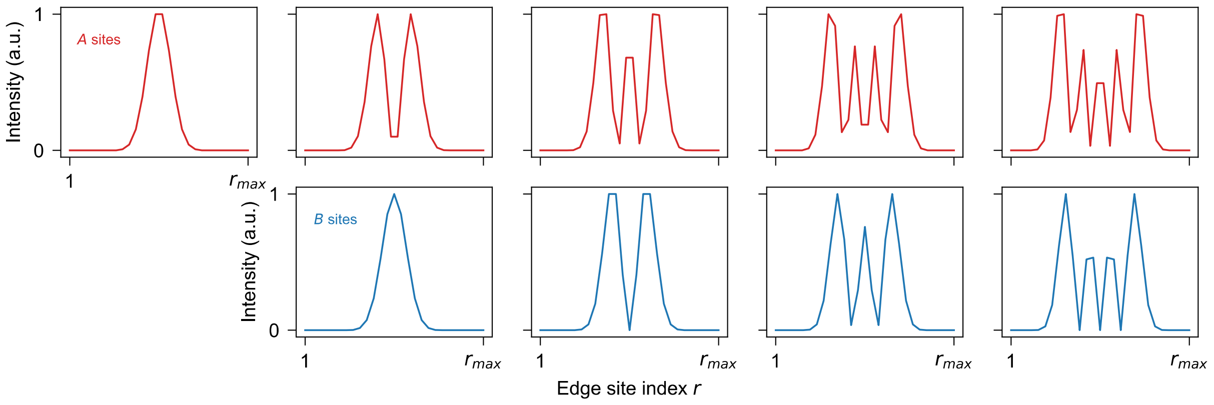

Figure S4. Same as Fig. S3, but for $N = 28$ (see also Fig. S2).

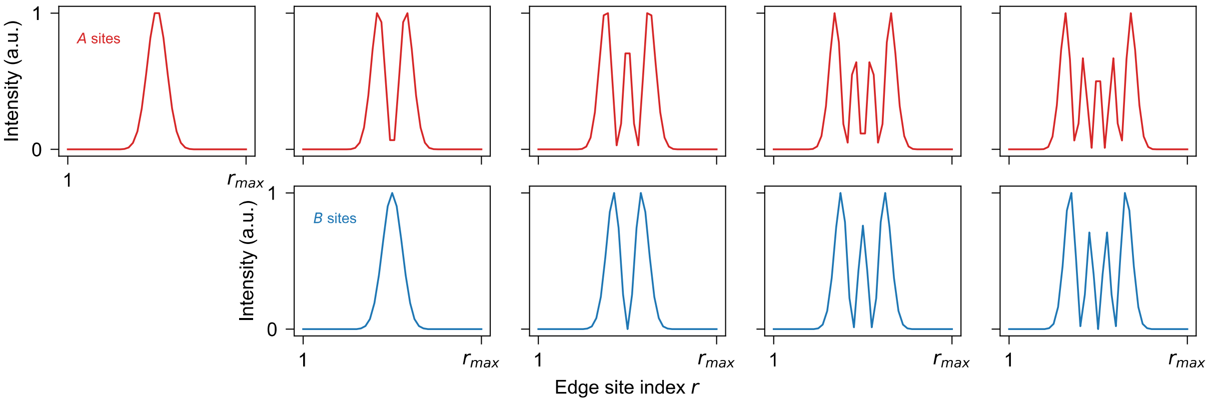

Figure S5. Same as Fig. S3, but for $N = 42$.

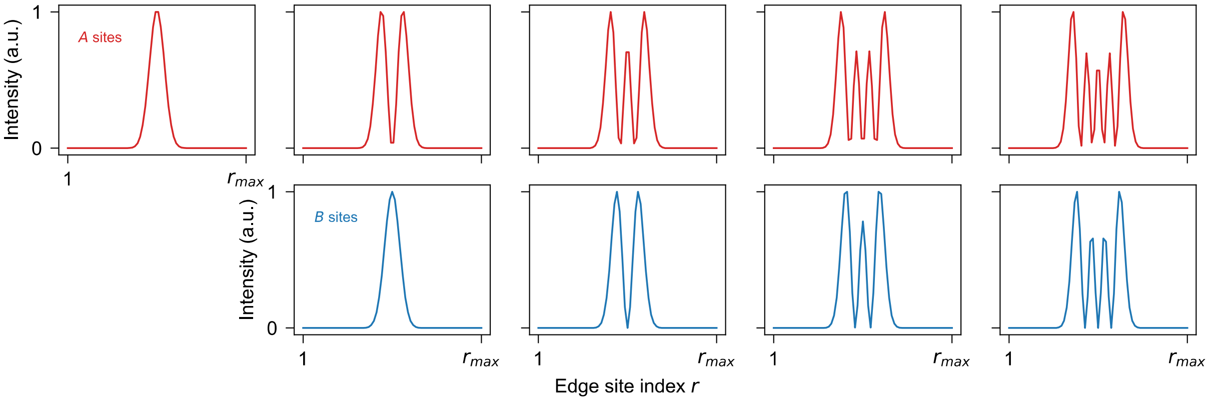

Figure S6. Same as Fig. S3, but for $N = 70$.

2. Analytical derivation of edge nodal patterns

We now present an explicit construction of these edge nodal patterns at maximal strain, which holds for any system size. We exploit that at this level of strain, the couplings can be rescaled uniformly so that they take integer values across the whole lattice[2,3]. Along the zigzag strand of a given edge, the couplings then follow the simple sequence

$$L-1, 1, L-2, 2, L-3, 3, \cdots, 1, L-1$$

In the zeroth pseudo-Landau level, an exact edge state along a single edge can then be found as

$$\psi_r^{(\mathrm{A,\,edge,\,0,}\,L)} = (-1)^r \binom{L-1}{r-1} \tag{S1}$$

where $\binom{n}{k}$ denotes a binomial coefficient, with all other amplitudes vanishing across the remainder of the system (we disregard overall normalization to keep expressions compact). We can now follow the procedure of Ref. 3 to iteratively construct the corresponding edge states in the higher pseudo-Landau levels. This is based on the explicit recursion relation

$$\psi_r^{(\mathrm{A,\,edge,}\,n,L)} = (r-1)\psi_{r-1}^{(\mathrm{A,\,edge,}\,n-1,L-1)} + (L-r)\psi_r^{(\mathrm{A,\,edge,}\,n-1,L-1)} \tag{S2}$$

between the A site patterns, as well as the direct relation

$$\psi_r^{(\mathrm{B,\,edge},\,n,L)} = E_{L,n}\psi_r^{(\mathrm{A,\,edge},\,n-1,L-1)} \tag{S3}$$

between the patterns on the A and B sites, with the weight factor $E_{L,n} = \sqrt{2Ln - n^2}$ equal to the energy of the pseudo-Landau level for these uniformly rescaled couplings. Note that these relations connect adjacent pseudo-Landau levels with indices n and $n-1$ in systems of size L and $L-1$. Writing the solutions in the form

$$\psi_r^{(\mathrm{A,\,edge},\,n,\,L)} = (-1)^r \binom{L-1}{r-1} f_r^{(n,L)} \tag{S4}$$

equation (S2) translates into the recursion relation

$$f_r^{(n,L)} = \frac{(L-r)^2 f_r^{(n-1,L-1)} - (r-1)^2 f_{r-1}^{(n-1,L-1)}}{L-1} \tag{S5}$$

which is initialized by $f_r^{(0,L)} = 1$, giving for example

$$f_r^{(1,L)} = L + 1 - 2r \tag{S6a}$$

$$f_r^{(2,L)} = \frac{(L-2r)(L-r)^2 - (L+2-2r)(r-1)^2}{L-1} \tag{S6b}$$

etc. In the limit of a large system $L \to \infty$, the rescaled functions

$$g^{(n,L)}(x) = (-2)^n L^{-n/2} f_{xL^{1/2}+L/2}^{(n,L)} \tag{S7}$$

then obey the simple limit

$$\lim_{\mathrm{L}\to\infty} g^{(n,L)}(x) = H_n(x) \tag{S8}$$

coinciding with the usual Hermite polynomials. Indeed, in this limit, Equation (S5) becomes the standard recursion relation for these polynomials, $H_{n+1}(x) = 2xH_n(x) - \frac{dH_n(x)}{dx}$. Noting further that the edge zero mode in Equation (S1) obeys the analogous asymptotics

$$\lim_{L\to\infty} \psi_{xL^{1/2}+L/2}^{(\mathrm{A,\,edge},\,0,\,L)} \sim \mathrm{e}^{-\frac{x^2}{2}} \tag{S9}$$

the edge states approach the sequence of bound states of a harmonic oscillator. Finally, according to Equation (S3), on the B sublattice the corresponding sequence is simply shifted by one pseudo-Landau-level index.

Supplemental References

1. https://pypi.org/project/tbee/
2. Poli, C., Arkinstall, J. & Schomerus, H. Degeneracy doubling and sublattice polarization in strain-induced pseudo-Landau levels. *Phys. Rev. B* **90**, 155418 (2014).
3. Rachel, S., Göthel, I., Arovas, D. P. & Vojta, M. Strain-induced Landau levels in arbitrary dimensions with an exact spectrum. *Phys. Rev. Lett.* **117**, 266801 (2016).
